# Selections and Abstracts

**Published:** 1917-05

**Authors:** 


### SELECTIONS AND ABSTRACTS

### THE HYGIENE OF PREGNANCY.*

**By W. C. Huessy, M. D.**

Seattle, Wash.

It is estimated that over 20,000 women die in the United States every year as a result of direct or indirect effects of pregnancy, labor and the puerperal state. In recent years, endeavoring to prevent complications and reduce the morality rate occurring during pregnancy, obstetricians are strongly advocating the more careful supervision of the expectant mother during gestation. Many important advances have been made in the management of pregnancy which have been far-reaching in their results for the welfare of both the mother and child, so that now it can safely be said that most of the complications of labor and the puerperium can be forestalled and the dangers materially lessened by proper prophylaxis.

Pregnancy from its incipiency renders a woman liable to toxemia. The border line between health and disease is less sharply drawn at this time; derangements so slight as to be of little consequence under ordinary circumstances may readily give rise to pathologic condition which may seriously threaten the life of the mother, child or both. It is because the changes in the maternal organism are so mainfold and the dividing line between the physiologic and pathologic so ill defined that it is particularly necessary to keep these patients under strict supervision and to be constantly on the alert thru the intervening months for the appearance of untoward symptoms in order to guide them safely to labor through the puerperium.

This can only be accomplished satisfactorily by giving these cases considerable time, care and consideration. The giving of directions as to personal hygiene, diet, sleep, exercise and dress is not all that is necessary but in addition upon the accoucher devolves the duty of seeing that a proper balance of elimination is being maintained, that all the physiologic functions of the woman are being kept at their highest efficiency thru the perfect elimination of waste material by the bowels, together with proper stimulation of the skin and the careful regulation of the intake of food materials. It is only by so doing that the diseases of pregnancy which may seriously threaten the woman's life or that of the child in utero can be and are averted. The accoucher ought to feel himself responsible for his patient's well being from the day she consults and engages him, and she should recognize that he has accepted the responsibility and feel that she is really under his care.

Ballantyne emphasizes the importance of careful history taken in these cases. A history of alcoholism, insanity, venereal diseases, hemophilla and hephritis will often shed light on many complications occurring in pregnancy and parturition and indicate what measures to pursue in preventing and treating such complications.

The kidneys being the most vulnerable point in the body during pregnancy, makes it very important that careful attention be given to the renal function. Of all the criteria of the condition of a woman during this period, none is so available or so trustworthy as the frequent examination of the urine. The careful analysis of the kidney excretion gives valuable information of the general metabolism. The urine should be examined every three weeks until the fifth month and then every week until term or oftener, if there is any reason to suspect trouble. The test should be made for albumin, sugar, specific gravity, the amount of urea, the degree of indican and microscopically for casts.

I consider it very important to ascertain the presence of indican and the degree of it exists in the urine in these cases. A persistent indicanuria is a forerunner of high blood-pressure. High blood-pressure is a more common and earlies sign of toxemia than albuminuria. If found present, the regulation of the diet is the first step. A purin-free diet will tend toward the elimination of indican, thereby removing one of the factors causing the complication of high blood-pressure.

These organs during pregnancy have more to do than in the non-pregnant state, as they are called upon to excrete waste products both of the mother and child. This excretion is favored by keeping the urinary tract well flushed out with large draughts of water. These patients should drink, if possible six glasses of water each day between meals, one before breakfast, two in the middle of the forenoon, two in the middle of the afternoon and one at bedtime.

The diet during pregnancy requires equally the same consideration as that given to the kidneys. Many of the ills that accompany parturition are brought about by improper diet and it is thru errors in diet that the most frequent cause of trouble arises. No inflexible rule can be given for these cases. Fair quantities of food are always needed. It is my custom to regulate the diet according to the reports of the urinalyses received from the laboratory. If there is no rise in the blood-pressure and the reports state albumin negative, a satisfactory nitrogen output and no indicanuria, a diet that will not overtax the kidney excretion, consisting of a moderate supply of nitrogenous food and a generous amount of vegetables and fruit, is advised. A good mixed dietary is the one best adapted for the pregnant woman. It should be plain, simple, easy of digestion, highly nutritious and partaken of at regular intervals. In many pregnant women, however, it is advisable to modify the diet. The necessity for this is shown by the presence of certain symptoms or physical signs which indicate the existence of a special strain on the kidneys and other organs of excretion. In these circumstances the lightest possible diet, one that throws the least strain on the kidneys and other glands, must be given. In severe cases it may be necessary to have recourse to an exclusive milk diet. In less severe cases, a diet of milk, bread, farinaceous foods and fruits may suffice, while in the milder forms of derangement all that is necessary is that the patient should avoid meats and richer dishes of all kinds, and live on a lactovegetarian diet. During the later part of pregnancy, when the gravids eturus has risen and presses on the stomach, greater care must be exercised in selecting the diet, carefully guarding against overloading the stomach by takink food greater in moderation and at shorter intervals. It is often at this stage that a milk diet is especially needed.

Too much importance cannot be laid on the necessity for the proper hygiene of the mouth and teeth. Early in pregnancy the teeth should receive careful attention by a good dentist, small cavities should be cleaned and filled and bad teeth extracted. Much can be done towards preventing their deterioration by relieving the acidity of the saliva through the daily use of alkaline mouth washes containing sodium bicarbonate or milk of magnesia.

In most women the bowels are commonly constipated, and pregnancy aggravates the disorder, intensifying the ill results accruing from incomplete intestinal elimination. This is due not only to mechanical obstruction but to diminished intestinal action as well. The bowels should be evacuated at least once daily. Regularity of habits and diet composed of articles of food having an aperient action, such as the cereals, coarse bread, vegetables and fruits, will often accomplish much in relieving constipation. Purgation is seldom called for. When medicine is indicated a teaspoonful of Husband's calcined magnesia in a glass of water, milk or lemonade or the compound rhubarb pill of the U. S. Pharmacapeia before retiring, I find are very efficacious.

Regular exercise in the open air should so far as possible be taken daily, its character depending largely on the previous habits of the patient. Moderate exercise can almost always be well borne. Long standing, excessive, or violent exercise or its undue prolongation must be avoided in all cases. Horseback riding, autoing over rough roads, heavy lifting and all violent muscular strain and overwork must be prohibited. Railway travel and ocean voyages should be postponed if possible. When for various reasons outdoor exercise cannot be taken, massage in the hands of a skillful person is to be recommended.

The dress should be simple, warm, loose and easy and shrould be so adjusted as not to exercise undue pressure upon the chest and abdomen. Garters and tight corsets should be discarded.

In multiparae with lax abdominal walls and in primiparae towards the end of pregnancy much comfort can be derived by supporting the lower abdomen with a properly constructed abdominal supporter. It must not be worn too light so as not to increase the pressure on the pelvic and renal veins; it should exert a lifting rather than a constricting pressure.

The pregnant woman requires an abundance of sleep, because of its health-giving, restoring influence. Eight hours daily of undisturbed sleep are essential. In addition to the night's rest, an hour or two either of rest or sleep after the noon-day meal should be encouraged.

The elimination through the skin of the woman during pregnancy is a process which certainly should not be neglected. The functions of the skin should be kept active by frequent bathing during the entire course of pregnancy and particularly so in the latter months when it is important to relieve the kidneys as much as possible of the extra work thrown upon them. The taking of a daily bath is always advisable; its temperature may be either warm or cool, according to the previous habits and to the season of the year. Extremes of temperature are to be avoided.

Varicose veins are especially apt to be troublesome in multiparae. To prevent their increase and to provide against the dangers of rupture, the treatment is the usual surgical one, namely, to keep the legs bandaged from below up and rest with the legs elevated.

The medical condition of pregnancy is always important to consider. Usually during this period the condition of the nervous system is of high tension frequently undergoes a change, at times excitable and irritable, at other times despondent and morose. To prevent these manifestations the surroundings of the pregnant woman should be such as will promote happiness and an even temperament. Unpleasant news, frights and physical shocks are to be avoided as far as possible. Reassuring influences are ever helpful. These caess should have a judicious amount of amusement, open air exercis and recreation.

The breasts and nipples require care preparatory to lactation and nursing, especially in primiparae is this important. These organs should be examined a month before labor when any abnormalities such as abrasions and fissures can be treated. If the nipples are at all depressed, the patient should be taught to draw them out gently with her fingers for a few minutes several times a day. In addition to forming the nipples they should be bathed night and morning with a soft cloth, warm water and soap and when dried they should be massaged for five minutes with solid albolene or sterile vaseline so as to make them soft and pliable. No strongly astringent washes should be used.

It is during the latter part of pregnancy when prophylaxis is productive of the best results. In order that the woman may be safely deliverel with due regard to her health and the being of her child, it is highly essential that conditions which call for a departure from the normal procedure be discovered six or eight weeks before the expected date of confinement, so that if necessary she can be placed under proper conditions to receive the help she needs. Careful pelvic mensuration and an abdominal examination should be made in every case at this time so as to determine the presence of any abnormality or existing degree of pelvic contraction. Ample time will then have been allowed to properly treat the case according to the findings presented. If an abnormal condition exists, its knowledge aids in advance in determining what course to pursue as to the treatment, whether to choose the induction of premature labor, to wait till term and depend upon the use of forceps, to resort to podalic version or a cesarean section.—**Northwest Medicine.**

---

### THE BRIEF STUDY OF THE PHILOSOPHY OF DR. OLIVER WENDALL HOLMES.

---

**J. S. Danforth Taylor, M. D.**
East Boston, Mass.

---

In the "rush and bustle of this practical, work-a-day world," when the mind of the busy physician is taken up with the duties of his practice, little time is found for reading much literature that is considered heavy.

Each one of us has our own particular hobby which we love to ride, such as photography, carpentry, mechanics, etc., with which we rest our tired brain cells and obtain much needed change and recreation.

I wish I could interest all medical men in the study and enjoyment of the biographies of some of the master minds in the medical world of the past and present.

To read such literature is to become intimately acquainted with those men and their names and their times, to obtain a broader view of life and to fasten in one's mind many important points in the development of medicine and surgery that would otherwise be neglected.

The philosophy and intellectual life of Dr. O. W. Holmes offers food for thought that should be appreciated by all medical men, especially those of Harvard training.

"Know old Cambridge? Hope you do—
Born there? Don't say so? I was too."

The date of Holmes' birth is given as August 29th, 1809. His father was a clergyman of the old school and Oliver had the advantage of being raised among books, and everything written by him shows traits of the student and scholar.

His course of reading included law, so that a man with such an all around education could not escape being considerable of a philosopher; and his far reaching views gave him profundity in almost every region of thought.

He lived in the clear atmosphere of the intellect, and relations far beyond his contemporaries were visible to his clear, penetrating vision.

Every woman in the world owes him a debt of gratitude, for he was the first man in America to call attention to the contagiousness of puerueral fever.

In 1842 he noted the fact that this disease seemed to follow certain physicians and at once began to study the cause of the infection. He made the remark that he "could tell what woman would be infected upon learning who had been her medical attendant."

To the few who escaped the disease he ascribed some individual immunity and this fact has been lately demonstrated to be true.

Many of the physicians of that time contended that the disease was due to the "Will of Divine Providence," but Holmes knew better and contended that the disease was due to some kind of miasma and was much pleased when, in later years, it was clearly proven that he had anticipated the bacterial origin of the disease, and that "dirty paws" and not any "divine Providence," was the advance agent of the trouble.

For the person who anticipates or introduces radical ideas, there is a Calvary in every twon and city of the world and nailed to its crosses of social ostracism may be seen the local criminals with the local genius in the center.

Holmes realized this, for his early investigations in regard to puerperal fever had brought upon his head the anathemas of both the medical profession and the clergy.

Those were the days when theology held full sway in our country, but thanks to the advancement of science, the older interpretations have become untenable and higher ideals based on scientific reasoning are sought after by a more enlightened race.

The works of Holmes have gone into every library in the world, but his freethought writings were so carefully injected into his works that it was some time before the clericals, so to speak, "got wise to him." Then they at once denounced him as a "Freethinker and subverter of Christianity," but so high a position had he attained in the scientific and literary world that "every knock was a boost."

In the "Autocrat" he tells us plainly that he is not a churchman and that he does not believe in "planting an oak in a flower pot." When we examine this book carefully we find a persistent determination to root out theology from the human mind, for the Gods of the past did not suit him. He was a deist only as he worshipped the God of nature, and all through his books we find contempt for ancient creeds and their propagators.

He was fully aware of the fact that religion had its origin in the sex-feelings and that fact is shown in his depiction of Myrtle Hazard and the Rev. Dr. Stokes.

The Pope comes in for his share of ridicule in the following verse:

"Who know's a woman's wild caprice?
It's played with Goethe's silvered hair,
And many a holy father's "niece,"
Has softly smoothed the Papal chair."

Someone told Holmes that his thoughts were dangerous, and he replied, "Dangerous to what? Truth will get well if she is run over by a locomotive while error dies of lockjaw if her finger is slightly scratched. The fear of discussion implies feebleness of conviction."

Some of his quotations are radical enough to suit the most rabid. For instance, "The aim and end of our free institutions is to think what we like and say what we think. Iconoclasm is rough but the only way to get at the truth. The whole system of beliefs that came in with the story of the fall of man is gently fading out of enlightened human intelligence and there will be no rest until legendary anthropology with all its dogmatic dependencies is given up. Democratic America has a different humanity than feudal Europe and so must have a new divinity.''

Had Holmes combined in one discourse these sentences which he scattered throughout his various books, they would have been rejected by every library in the country.

Said Holmes one day to a friend who had warned him to be careful how he talked about religion, ''Religion and Government appear to me the two subjects which of all others should belong to the common talk of people who enjoy the blessings of freedom. I do not see anything so fit to talk about or half so interesting as that which relates to the destiny of our fellow creatures.''

And Holmes was right. Living in a country where freedom of speech and conscience are the very fundamentals of our constitution, what subject is there that cannot be freely investigated and subjected to scientific criticism?

Says Holmes, ''It is not science alone that Christian pessimism has to struggle with, but the instinct of childhood, the affections of maternity, the intuitions of the poet, the humanity of the philanthropist, in short, human nature and advanced civilization.''

''We cannot have self government and humane laws without a complete reaction in our religious views.'' ''We frequently see persons in insane asylums and hospitals, sent there in consequence of what are called religious mental disturbances. I confess I think better of them than of many who hold the same notions and keep their wits and appear to enjoy life very well. Any decent person ought to go mad, if he really holds such opinions.''

Again Dr. Holmes says: ''A French physiologist once confined tadpoles under water in the dark. Removed from stimulus of light they could develop neither legs nor arms. They swelled and grew and grew into gigantic tadpoles. I have seen hundreds of colossal human tadpoles and a considerable number of the Holy Father's one hundred and thirty-nine million of followers are overgrown larvae, sculling about in the dark by the aid if mere caudal appendages.''

Such is the comment of Dr. Holmes on the Christian religion.

That he was an agnostic and a freethinker no one can doubt after reading his works; and as a matter of fact, can the intelligent physician, who has access to all modern literature, backed by his own science, be anything else?

And what an opportunity the physician has in his profession, to help drive out fear and superstition from those with whom he comes in contact and to assure them of the natural order of the universe?

In poetry Dr. Holmes excelled as in anything else and it is in his humorous verses that he pleases most.

Who has not read ''Old Ironsides'' without a renewed patriotic feeling and who has not smiled at the ''Deacon'' and his ''One Hoss Shay?''

No medical man should fail to read ''The Stethoscope Song'' and he who can read ''The September Gale'' without cracking a smile for the rest of the day is indeed devoid of humor.

In rhetoric he was the master of metaphor and in ''The Chambered Nautilus'' he is in close rivalry with Robert Burns in the use of his similitudes.

''Elsie Venner,'' ''The Autocrat of the Breakfast Table,'' and in his poems the memory of Dr. Holmes will never die.

If this brief paper may stimulate someone to learn more of this great man, physician and author, and to encourage the realization of the value of biography. its object will have been accomplished.—**The Medical Times.**

### WAR.

After many months of discussion and of efforts to avoid hostilities the Government of the United States has been compelled to admit the existence of a state of war with the German Government. In the moderate, measured, but forceful address of the President of the United States to Congress, it is clearly pointed out that the American people have no quarrel with the people of Germany, but only with the Government which has overborne American rights on the high seas, has sunk American vessels, and has killed American citizens. Much as all thinking people deplore war and all that war means, there can be no question but that the people of the United States as a whole are convinced that the time has come when the existence of a state of war must be acknowledged, when they must begin to resent the attacks to which they have been submitted during the past two years.

It is most unfortunate that these two years have not been devoted to an active preparation for an eventuality which the majority of the Americans have long foreseen. We have learned much by observation of the trials which England has gone through in organization for war. Our position is quite analogous to that of Great Britain. We have, within its limits, an efficient navy, we have an excellent and well trained regular army, though, like that of Great Britain, it is a very small one, but beyond this we are totally unprepared. We have no trained reserves to fall back on. We have to contend with all the faults inherent to democracy at war. We lack the centralized authority, the one man power which makes an autocracy such an efficient war machine. We should profit by the example set by the British and early accord to the President the full measure of authority just as he must bear the full measure of responsibility for the conduct of the war.

War cannot be conducted in any halfway manner. We must bring to bear every ounce of energy, every drop of blood, and every dollar that we possess, and must have this directed by the best brains in the country that this bloody business may be brought to an early and successful close.

Our regular army now numbers 125,000 men, and the National Guard about 160,000. If recruited to full war strength they would number 280,000 and 440,000 respectively, or a total of 720,000. For this army some 5,000 medical officers would be required. In addition to this we have another army of 500,000 called to the colors for training, making a first line defense of 1,220,000 men. In addition, it is expected that at least a million and a half troops will be called out during the year for training, making a total of 2,720,000 troops under arms. Such an army would require 19,000 medical officers. We now have in the army about 1,200 medical officers, and in the National Guard about as many more, making about 2,400 officers, and leaving a deficiency of 16,600 medical officers to be supplied from civil life. In view of the fact that we have in the United States only 145,000 registered physicians it will be seen that the new army will face a deficiency in a direction in which it will be difficult to improvise aid.

We have repeatedly published the appeal which has been made for medical reserve officers. That appeal still stands, and very qualified medical practitioner should weigh well his obligations and decide whether he is in a position to offer his services to his country and help fill this deficiency in a most important arm of the service.

Besides the need for medical reserve officers, there are at present 230 vacancies in the medical corps of the regular army. Under the staff bill for universal service 1,811 additional medical officers would be required for the permanent establishment, making a total of 2,041 opening for appointment in the permanent force. Here is a very attractive field for young physicians who have had the required year of hospital experience. Steps are already being taken by some of the State and county medical societies to safeguard the interests of those practitioners who are willing to give up their practices temporarily for service in the army. This work should be pushed rapidly and organization effected, so that those who wish to serve their country can do so with the least possible pecuniary loss.— New York Medical Journal.

---

### THE PRESENT STATUS OF MOVING PICTURES IN SURGERY AND MEDICINE.

**Orrin S. Wightman, B.A., M.D.**

At the present time moving pictures have taken so firm a hold upon the public, that this method of presentation has impressed the medical profession with its value as a teaching medium.

In earlier days eht work was somewhat crude, owing to the fact that the operator did not realize that certain technic was necessary to produce a smooth-running picture. The advantage of working slowly and postively, keeping the field clear for the camera, replacement in position where a picture was interrupted, the value of using proper artificial light, which properly illuminated the fieldfi and avoiding as far as possible the deep shadows and blackened whites of the skin—all these factors in the achievement of good results have been attained only after painstaking care and careful cooperation on the part of the surgeon, the camera man, and the director.

Without a technical knowledge of the possibilities and limits of photography, many have failed to realize that in black and white work the skin, which is pink, is not a constant white, but prone to take the dark tinge of the reds, while blood takes on all the negative phases of black. Again the shiny surfaces due to the reflection of wet areas invariably tend to intensify the impenetrable black of the reds. For these reasons, if moving pictures are to accrately visualize the motions of the operator, he must realize that the camera cannot follow the finger, and must content himself with portrayiny only what is comparatively superficial. If many of the camera men realized this, we would doubtless be spared many feet of film which are absolutely useless, beside being trying on the eyes of a wearied audience.

While the present tendency of surgical and medical moving pictures has drifted somewhat toward the spectacular, it is well to bear in mind that merely going through the technic of an operation is tiresome and becomes monotonous. Operations must be humanized. The patient is human, and the preliminary steps which are a part of an operation, including taking the patient to the operating room, the administration of ether, etc., prepare the audience for human things. The patient then becomes an entity, and the rapidly flying fingers in the subsequent procedure have a definite meaning.

The commercialized moving picture producers leave no stone unturned to produce this very result, and if handled carefully, there can be no medical objection.

There is a field even now for the surgical scenario writer who has an exact and carefully studied technic, which he can offer to any competent operator to carry out.

All of this may be objected to on the ground that we are departing from the strictly scientific presentation of surgery and entering the field of entertainment and instruction. This we freely grant. We believe this is a very live field for moving pictures.

After viewing many surgical moving pictures and doing some thousands of feet of medical film personally we are honest enough to admit that the average audience carries away impressions of rather an indefinite character and far different than the operator ment to convey. The picture presented is referred to as wonderful, etc., but when the spectator is pinned down as to what he actually learned in a scientific way, he confesses, if he is honest, very little.

This of course applies to the scientific and what is wrongly credited with being very technical. Again I repeat that the same picture after humanizing might not only be technical but instructive. Where steps in an operation are to be taken within the vision of the audience they must be taken rapidly and slowed down in projection, so that some mental impression of the procedure can be retained. We know that sixteen pictures are the actual number of changes in motion per second that are employed in motion photography. No one has yet stated the rapidity and retention of impressions of this character which are actually assimilated and retained. Only a fraction of what is seen is remembered, and it is surprising even to one constantly working in this field to realize how many new things are observed in each reshowing of the same picture. A picture can only be appreciated when seen a number of times and restudied. This may explain why so many in an audience are able to retain so little of the scientific and so much of the spectacular. If this observation is correct, we must take the people as we find them, and plan our work accordingly.

The student in the medical school is wonderfully benefited by observing the sequence of procedure in operations. This applies to what can be visualized. The true value in technic can be gained in no other place but the operating room, and then at the side of a teacher who impresses his various steps upon the student and lets each procedure sink in. What takes an hour in the operating room, may be crowded into ten or fifteen minutes in a reel— it is small wonder that confusion may result. Unless the operator stands beside his screen when the pictures are projected and explains the various steps in his operation, much may pass unnoticed or unappreciated. On the other hand, long titles are either trying on the eyes, or so long that an audience refuses to read them.

It is far easier to visualize general motion than to give in clear detail the work on an operative field. An audience tires of the concentration, and for this reason becomes fatigued more easily than where the motive is more apparent. On the other hand, where motion is of major signifiance and the idea to be presented is simplified, we find a most instructive field. Postoperative results, with renewed use of the parts, the gaits of the ataxic, the various methods of medical dianosis involving motions, etc.,—these lend themselves to visualization.

Moving pictures have come to stay. Let the medical profession line themselves on the side of progress, neither carried away by the possibilities of self-exploitation nor, on the other hand, losing the teaching possibilities by too much of the spectacular . Let us steer a middle course, which shall be not only instructive and scientific but well-staged as well.—International Journal of Surgery.

---

### NEUROSES IN RETURNED SOLDIERS.

By Goldin W. Howland, M.B., M.R.C.P. (London)
Neurologist to Out-Door Department, Toronto General Hospital.

The nervous cases that have passed through the hands of Dr. Julian Loudon and myself at the Military Convalescent Hospital show at the present time 75 per cent. functional nervous disorders as compared with 25 per cent. organic cases, a proportion which probably depends on the fact that many of the nerve injuries pass, I suppose, directly into the hands of the surgeon.

These functional nervous cases may briefly be divided into five divisions for the purpose of classification, but not, as I shall later show, from the standpoint of understanding them, and these divisions with their percentages are:

Neurasthenia, 59 per cent.
Hysteria, 21 per cent.
Epilepsy, 12 per cent.
Tic, 4 per cent.
Dementia praecox, 4 per cent

One can quickly dismiss the last class, as they fall into the asylum group, to which most of the insane cases go without entering our clinic. Of these we have seen it was evident that the war service was only a factor in increasing their natural course in life, and a majority of these cases never saw the battlefield, while those that did were unquestionably much the worse for the strain.

The **epileptic division** are of considerably more interest, as they naturally are given a certain pension. In our experience nearly all these cases were old chronic epileptics who should never have enlisted, or cases that had epilepsy and had been clear of attacks for a varying number of years. The result of service has been to cause a return of their attacks or a marked incease in their frequency, but fortunately many of these men were detected in their training. It is most advisable that a special outlook should be taken by medical examining officers for this disease, as the number who have enlisted and the total amount of their pensions must have cost the country a fair sum of money.

There are a certain number of epileptics produced in this war, but they appear mainly to be cases who have received true organic injuries from penetrating wounds of the brain and fracture of the skull.

The remainder of the cases forming 48 per cent. of the whole fall into the classes called neurasthenic, hysteric and tic conditions.

Now, I desire to lay great emphasis on the following facts: In reviewing the great number of symptoms presented by these soldiers, one finds it impossible to divide them into definite diseases, **i.e.**, neurasthenia, etc., but it is necessary to realize that their symptoms are disturbances in the functions of their nervous system, and that the same individual may have at the same time increased irritability in some function, exhaustion in another, and the typical dissociation of hysteria in a third. This is true not only for war cases, but also in civil life, and I firmly believe that it will be a great advance in the study of these disturbances if we look at them entirely as definite disturbances in function and not as separate and definite diseases. This method is followed in this paper.

Turning our attention in the first place to the psychical symptoms, one finds that insomnia is by all means the most common, and was a striking sign in 30 per cent. of patients, while in those who were able to sleep there are frequently dreams of the most nerve-racking type, so that they will rise from slumber in fear and trembling, and be glad to awake. This undoubtedly increases their general nervous condition, and must be controlled by hypnotics.

Many of the men state that they are exceeding irritable, and that they themselves are conscious that they are unable to control themselves as formerly, and if irritated they say it is at times difficult to think, but that they at once become excited, and cannot control their actions. Now this if of great importance, as there have been several disturbances of moment in certain places, and I firmly believe that it will be necessary for those in judicial places to carefully remember the characteristic that these men not only describe but heartily regret and ask aid for.

A secondary feeling arising from this is a feeling of restlessness, and it may be a source of difficulty in finally getting them settled at work, as indeed we have already found. This is not discontent, but a positive loss of the ability to remain quiet, even if occupied, and if forced to do so, ending in an increased degree of nervousness. No less than 25 per cent. of cases find that their memory is no longer good, and 8 per cent. of them have a definite dissociation of memory, of which this is a typical case. Private M., after shell shock, continued in the fighting line, but his comrades had to prevent him from marching away towards the enemy. On observation, he was found to have lost all memory from the time of his shock, excepting the knowledge of eating, etc., which no Canadian man or officer ever forgets. On arriving home, he, as follows, described his sensations "I got off the train and some woman threw her arms around me and kissed me, and a girl kissed me, and they took me home. Well. they said that they were my mother and sister, and I took their word, but they might be anybody's mother and sister for all I knew. I never saw the home before, and they took me to see where I worked, but

I never remembered seeing the place or the man in charge before." We have had several cases like this and all are doing well.

The perception faculties are naturally influenced by these same irritable conditions; in fact, this firing up on slight provocation can be regarded from this standpoint, so that we may expect that the soldier so affected will not be so apt as formerly at understanding correctly what is said to him.

I have not noted many cases in which the common disturbances of perception, seen frequently in civil cases, is present, namely, fears concerning the outside world, nor the equally common type characterized by fear concerning, and concentration of the mind on the viscera, i.e., the pschosomatic class.

The associative powers are parallel in their affection to the perception. Again, it must be noticed that a small percentage complain to exactly the opposite condition to this excess irritability, and state that they feel stupid and torpid and unable to think quickly, and are not easily aroused. Such a state corresponds to an exhausion of their mental faculties, and yet is associated with irritability or dissociation in other functions.

The emotional power is not affected as much as in civil practice, and I have only noted one or two cases where loss of control over crying or laughing was present.

Concentration and volition are parallel in their defect to the other signs, and while they are frequently noticed, yet they are seldom directly complained of.

The marked irritability of the cortex is shown by one symptom that is present in over 15 per cent. of cases, and is one of the hardest to cure, and that is dizziness.

It is found associated with all types of cases, and the difficulty in diagnosing it from the similar symptoms in epilepsy is constantly occurring. It is quite a different condition from the usual faint and giving-way conditions that are seen in ordinary nervous patients. It is parallel to that feeling we all get if struck on the head. It corresponds, therefore, to a condition in which the patient is on the verge of becoming unconscious and is due to extreme irritability of the brain and is not to my belief of vaso-motor origin.

Naturally it is the dividing line between control by the individual and the commencement of loss of control, and the resultant dissociation that must follow. The attacks may last a few minutes to hours and days, and occur once or more often a day, to once a month or less. It is important as the men are much hampered by it.

It is the crossways symptom from which one passes to irritability, to dissociation, and to epilepsy.

Naturally, from this symptom one passes to true attacks of dissociation or hysterical attacks proper, and in our record they are present in only a small number of cases.

Turning now to the disturbances in the psycho-motor and motor fields, tremor is by far the most common sign, and is present in 33 per cent. of cases, while if to these is added the symptom of shakiness, it is increased to 46 per cent.

This shakiness is, however, often used to refer to an exhausion condition, and in this connection should not be considered as a tremor and must be differentiated.

Tic of the face is an occasional unfortunate disturbance, and generalized tic of the body occurred in two cases, some 5 per cent. taking home this memory of the war. The results of treatment have been good, and I think all have recovered but one.

In regard to true motor dissociation, as seen in so-called hysteric paralysis, we have a record of 16 per cent. of cases, a truly high proportion for a condition formerly almost a prerogative of the female sex. What it would have been if the women went to war would have been a world that would have been paradise to a Charcot.

The cases here have been nearly all monoplaegias of leg or arm, but the associated hysterical anaesthesia has been usually on both limbs. They are the most difficult to cure as they have been worked at for a year abroad, and the failure there has sent them home. I think that it would be better if all functional cases were at once sent back to Canada instead of staying in the varied homes in the Motherland.

The psycho-sensory and sensory symptoms are very varied indeed, but may be classed under very few heads when they are collected. Headache is a very common one and takes all forms in the 20 per cent. cases with it, and very often is described as a pain in the back of the head, made worse by worry.

The common psycho-sensory symptom of extreme susceptibility to noise is comparatively rarely accomplished of but evidently much felt.

From pain in the head to pain in the back is a regular everyday channel, but while one finds 7 per cent. of the latter, yet it is far more frequent to find shooting pains in the legs, as double the number of cases have this and suffer severely from it.

Dissociation of sensation is reported, as anaesthesias in 15 per cent. of patients, and it follows the usual functional types, but one must be very careful not to forget that it is often found in organic disease also. Loss of visual sensations and of taste, smell and hearing are to be found in different clinics, and particularly the former is quite common.

When we turn to the sympathetic nervous system there are three outstanding symptoms. The first of these is sweating, and the 20 per cent. of cases with this are very striking, as in some the sweat pours off them to actually form little pools of water on the floor. Associated with this is extreme vaso-motor irritability, and tache cerebrale is very marked and very long-standing in its duration.

The third sign is rapidity of the heart's action, and unfortunately one must exclude the question of too many cigarettes and of possibly Graves' disease. The cigarette sickness is a fairly common cause, and many of the returned soldiers show the yellow finger but never the white feather.

Finally, one may add that vomiting, diarrhoea, shortness of breath and frequent micturition complete the picture of the varied functional disturbances in the returned soldiers.

In conclusion, a few words in generalizing the treatment of these cases. After these soldiers have undergone nine or more months under the care of the hospitals in England and France, they have practically had all forms of up-to-date treatment. I am in favor of their trying a period of three months in their homes where these are suitable, and then, if there is no improvement, taking them into hospitals and trying again to aid them. While in their homes it is best to give them such treatment by massage and bath as seems to help them.

Under this plan of treatment many of the cases are cured, and many have gone back to work, in fact it is often best to send them back as a means of treatment, and if not a success begin again their hospital course.

But there are a group of cases that the home treatment does not cure, and they require hospital care, as well as cases that get steadily worse at home in the three months there.

Now, the present convalescent hospitals are the worst places for these men, and they do better in the general hospitals.

There must be a home for these nervous cases established apart from the military hospitals, and it is also necessary that this must not be used for mental cases, as the combination is bad indeed. It should be near a big city and be used only for this class of cases and for those whose homes are not suitable. I would suggest that it be a central hospital for all the adjoining districts.

It would also be very valuable for cases in their first home treatment if there was some plan to board them out at Muskoka and Georgian Bay Points, as the cases going up there do most extremely well.

So that in conclusion I advise (1) Home treatment, 3 months; (2) If no improvement, or gradual decline, a stay in a nervous hospital; (3) Arrangements for improving cases to go north; (4) Testing improved cases at work.— Canadian Practitioner and Review.

